# Black Esophagus: A Life-Threatening Consequence of Hypoperfusion

**DOI:** 10.7759/cureus.75769

**Published:** 2024-12-15

**Authors:** Jihane Benass, Sanaa Berrag, Chaimae Jioua, Salma Ouahid, Hassan Seddik

**Affiliations:** 1 Gastroenterology II, Mohammed V Military Teaching Hospital, Rabat, MAR; 2 Gastroenterology I, Mohammed V Military Teaching Hospital, Rabat, MAR

**Keywords:** acute esophageal necrosis (aen), black esophagus, esogastroduodenoscopy, gi bleeding, hypoperfusion

## Abstract

Acute esophageal necrosis (AEN) is an uncommon endoscopic finding characterized by a patchy or diffuse circumferential black pigmentation of the esophageal mucosa, corresponding to ischemic necrosis. It usually presents with upper gastrointestinal bleeding and is thought to be caused by a systemic low blood flow in patients with predisposing risk factors, like advanced age and cardiovascular comorbidities. After initial hemodynamic stabilization, diagnosis is established by esophagogastroduodenoscopy (EGD) with careful biopsies and histological evaluation. Delayed diagnosis is thought to be the main cause of panesophageal necrosis, perforation, mediastinitis and septic shock.

We present a case of AEN triggered by hypoperfusion due to severe perioperative blood loss in a 72-year-old woman with a history of cardiovascular disease. Even though she was stabilized during surgery, she still presented with melena 24 hours after, and endoscopic examination showed ischemic patches consistent with AEN. After administering intravenous fluids, proton pump inhibitors (PPI) and parenteral nutrition, a repeat EGD after 72 hours showed a neat improvement with erosive and ulcerative lesions replacing the black patches and a complete healing after eight weeks of oral PPI therapy.

AEN is a rare but highly deadly entity that endoscopists must be aware of, especially in patients with multiple comorbidities and presenting with gastrointestinal bleeding after an episode of hemodynamic instability. Quick management can significantly improve survival.

## Introduction

First described by Goldberg et al. in 1990 [1], acute esophageal necrosis (AEN) or ‘esophageal stroke’ or ‘black esophagus’ is a rare endoscopic finding secondary to a systemic low-flow state. It is characterized by a patchy or diffuse circumferential black coloration of the distal esophageal mucosa stopping at the gastroesophageal junction and occuring without the ingestion of corrosive or caustic substances [2-4].

We report a case of black esophagus successfully treated with aggressive resuscitation and intravenous (IV) proton pump inhibitors (PPI).

## Case presentation

A 72-year-old woman presented with melena 24 hours after a radical cystectomy for muscle-invasive bladder cancer. She had a history of hypertension and cardiovascular disease but no history of alcohol abuse or diabetes mellitus. The surgeon reported a significant perioperative blood loss but the patient was transfused and was stable after surgery. On examination, she was tachycardic at 110 beats per minute, hypotensive at 90/50mmHg, afebrile, with a percutaneous oxygen saturation of 98% on ambient air. Laboratory data showed a hemoglobin level of 7 g/dL, a blood urea nitrogen level of 12 mg/dL, a creatinine level of 0.64 mg/dL and a fasting blood glucose level of 110mg/dL (Table 1).

**Table 1:** Laboratory findings

Parameters	Patient values	Reference range
Hemoglobin	7g/dL	12-15g/dL
Blood urea nitrogen	12mg/dL	5-20mg/dL
Creatinine	0.64mg/dL	0.6-1.1mg/dL
Fasting blood glucose	110mg/dL	70-100mg/dL

**Figure 1:**
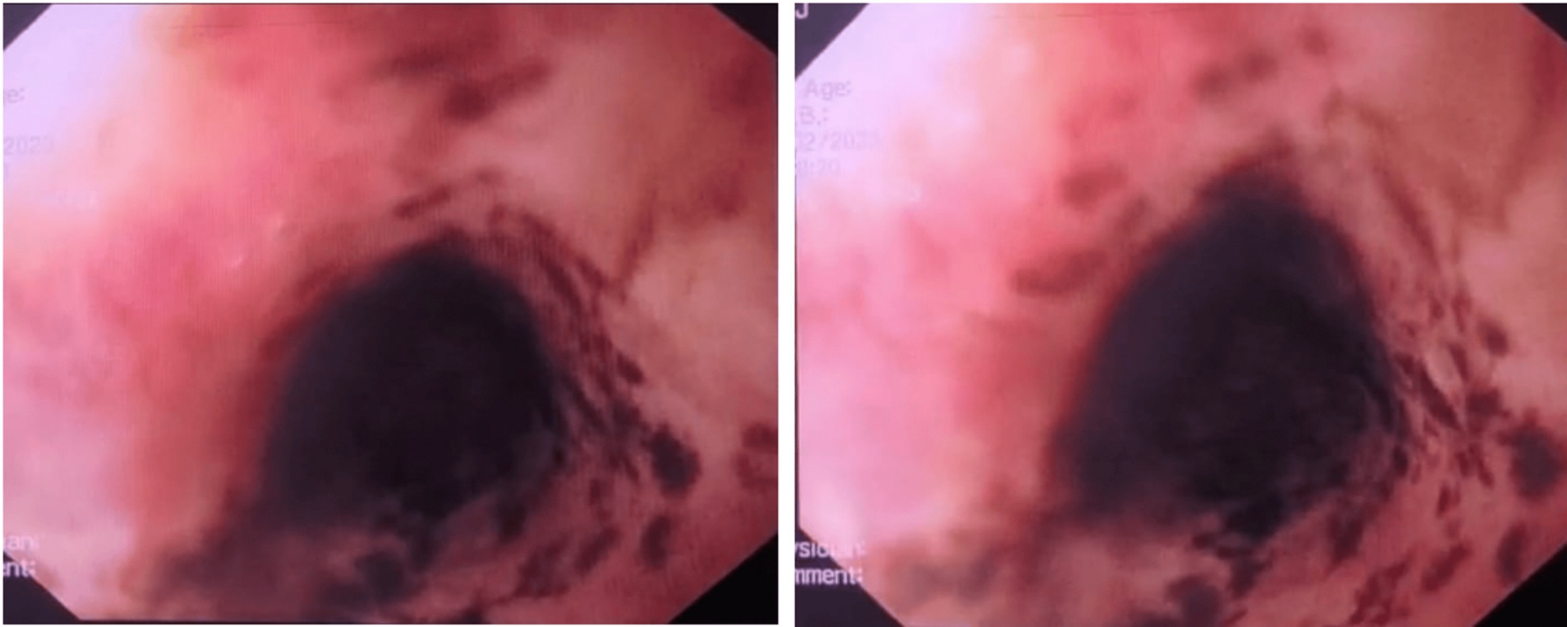
First esophagogastroduodenoscopy with patchy necrotic mucosa: detail of the middle third of the esophagus

**Figure 2:**
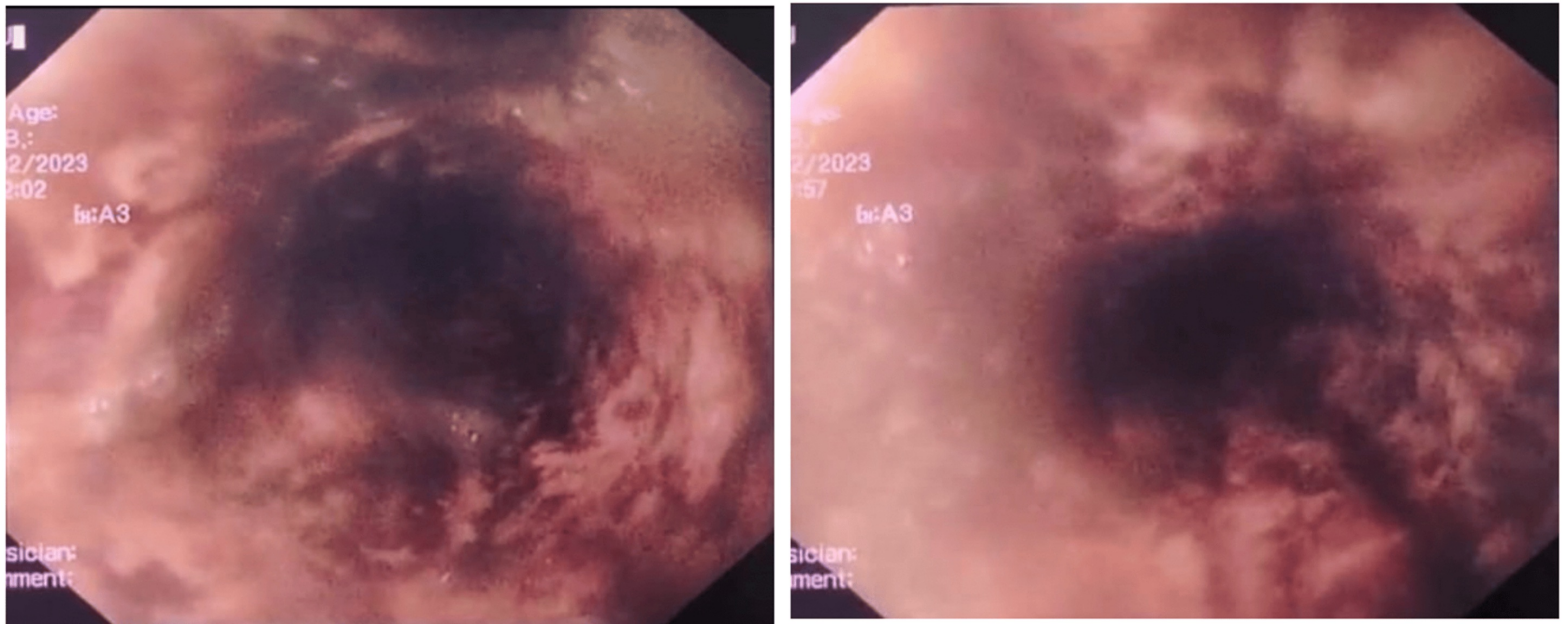
First esophagogastroduodenoscopy with patchy necrotic mucosa: detail of the distal third of the esophagus

**Figure 3:**
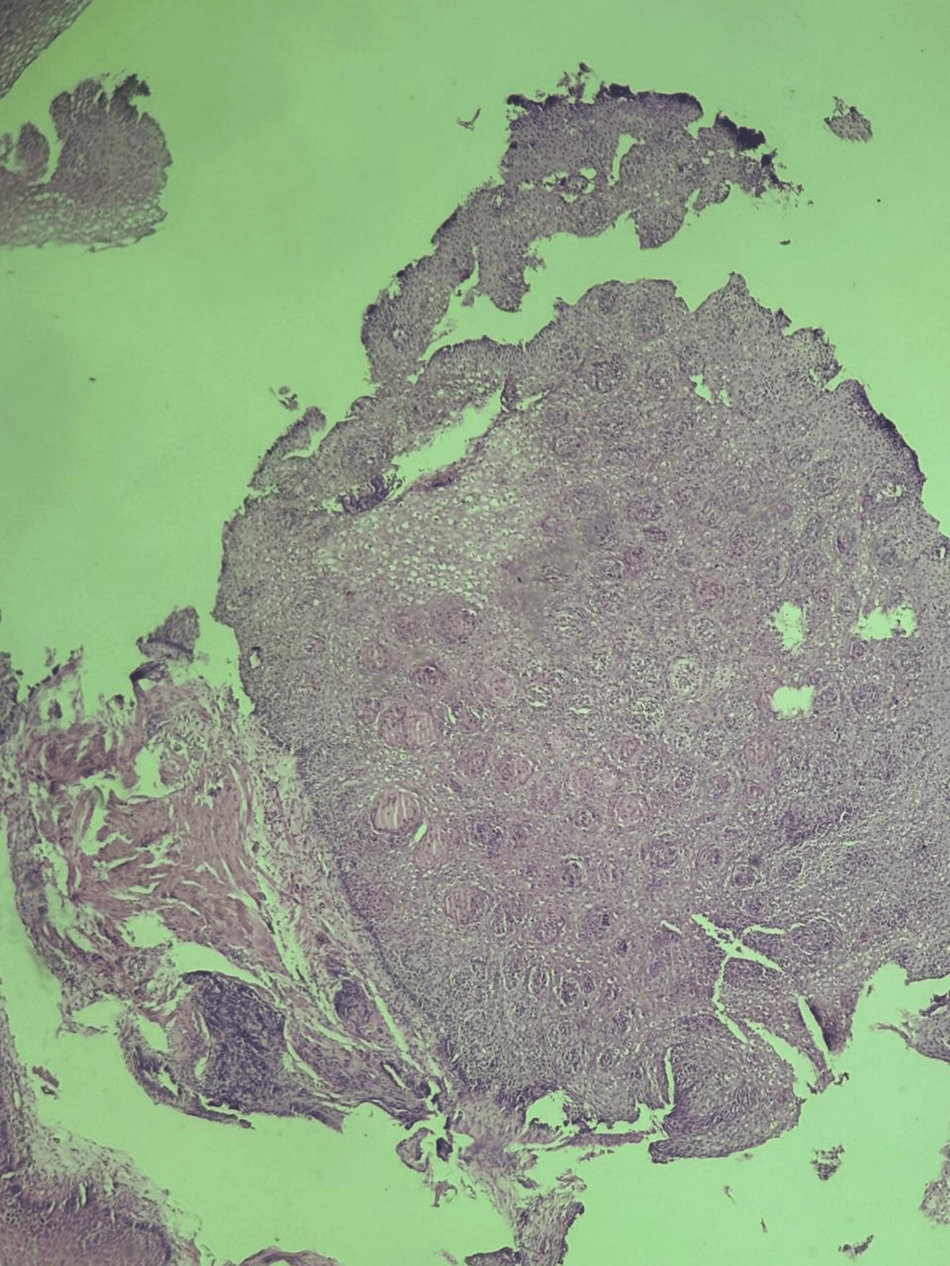
Biopsy results showing ulcer exudate and granulation tissue with acute inflammation

She was started on aggressive intravenous proton pump inhibitor therapy and an esophagogastroduodenoscopy (EGD) was performed after transfusion of two units of packed red blood cells, approximately 10 hours after the first occurrence of melena. The endoscopic examination showed normal mucosa in the upper third of the esophagus, while the mucosa in the middle-lower third appeared black (Figure 1 and Figure 2). This aspect did not extend beyond the gastroeosophageal junction, with a clear demarcation. The biopsy results showed ulcer exudate and granulation tissue with acute inflammation, with necrotic changes in the mucosa no indication of fungal presence, nor cytomegalovirus nor herpes simplex virus (Figure 3).

**Figure 4:**
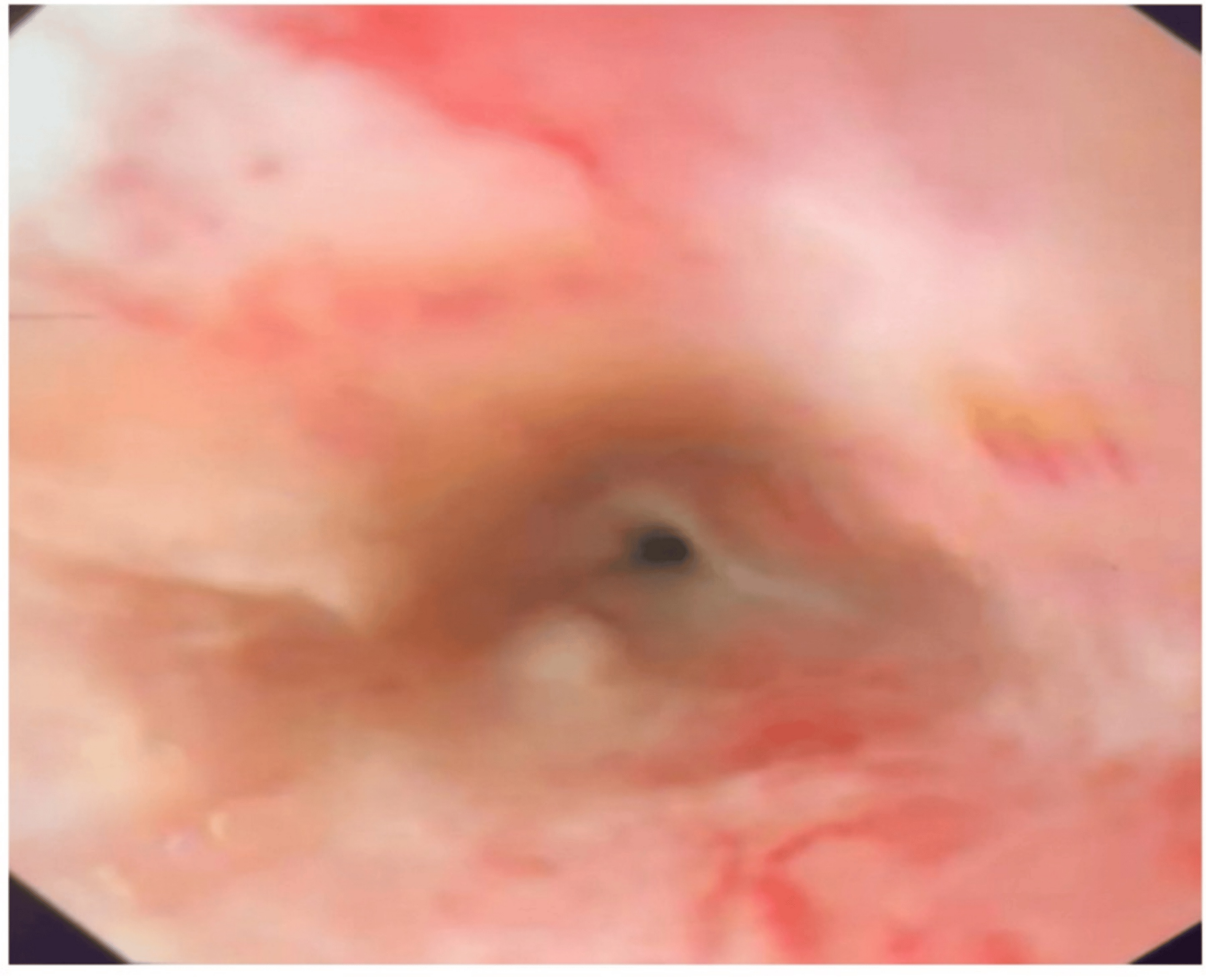
Follow-up esophagogastroduodenoscopy (after 72 hours) with close to total healing of mucosal necrosis

Parenteral nutrition was started and high doses of IV PPI were maintained while strict bowel rest was enforced. A repeat EGD was performed 72 hours after the first one and revealed healing with a neat improvement in the blackish discoloration with multiple ulcerative and erosive lesions and white excudates (Figure 4). The patient had an uneventful recovery afterwards and was discharged in a stable condition under oral PPI. Total healing of esophageal necrosis was observed at the eight-week follow-up esophagogastroduodenoscopy and she did not develop esophageal stricture.

## Discussion

AEN is an uncommon endoscopic finding caused by systemic low blood flow manifesting as a patchy or diffuse black appearance of the distal esophageal mucosa, typically halting at the gastroesophageal junction, and occurs in the absence of corrosive or caustic substance ingestion [2-5].

Its incidence is estimated to range between 0.01% and 0.2%, with less than 200 cases reported in the literature [6]. A study conducted on 310 consecutive autopsies has demonstrated a 10.3% incidence of AEN, suggesting it is an underdiagnosed condition [7].

It is thought to arise from the temporary reduction in esophageal blood flow. Decreased perfusion can quickly result in extensive esophageal necrosis, which can be potentially reversible upon restoration of blood flow. Maintaining satisfactory blood supply to the esophageal mucosa, lamina propria, and submucosa is fundamental for preserving cellular function and protective barriers. When esophageal blood flow is compromised and reperfusion injuries occur due to reactive oxygen metabolites, both direct and indirect damage to cellular defense mechanisms ensues, leading to cellular breakdown and necrosis. The distal third of the esophagus has a poorer arterial blood supply provided by small branches of the left gastric artery. The segmentation of its arterial network makes it particularly susceptible to ischemic injury [6,8-10].

Moreover, case reports have noted associations between AEN and viral infections, gastric volvulus, antibiotic hypersensitivity, Stevens-Johnson syndrome, hyperglycemia, diabetic ketoacidosis, lactic acidosis, and trauma [4-6,10].

Risk factors include male sex, advanced age, typically the sixth decade of life, cardiovascular disease, alcohol abuse, diabetes mellitus, diabetic ketoacidosis (also called Gurvits syndrome), malnutrition, hiatal hernia, malignancy, chronic kidney disease, and chronic pulmonary disease [4,5,8,10,11].

In a large systematic review of 130 patients, patients with AEN mainly presented with upper gastrointestinal bleeding [10]. In rare cases, symptoms like dysphagia, epigastralgia, vomiting and syncope have been described [4,6,8,10,12].

Following initial stabilization, the positive diagnosis of AEN is made during endoscopic evaluation and histological examination of esophageal biopsies. The typical EGD finding is a widespread circumferential discoloration of the distal third esophagus with a clear transition to normal-looking mucosa at the Z-line, though cases where the upper part or the entire esophagus was involved were reported [5,6,10]. The extent of esophageal necrosis seems to vary based on the severity of the disease and the duration between symptom onset and presentation. Biopsies should be performed carefully due to mucosal fragility.

The differential diagnosis encompasses conditions such as melanosis, pseudomelanosis, malignant melanoma, acanthosis nigricans, coal dust deposition, and necrosis due to ingestion of caustic substances [2,4,6,8,9].

Currently, the available evidence for treating AEN is sparse. Early identification and hemodynamic stabilization through aggressive resuscitation are key principles in managing AEN and contribute to improved disease outcomes [6,10].

IV PPI with antiemetics, total parenteral nutrition and nothing per oral are important therapeutic resources. Physician awareness is also crucial since early recognition and prompt management can improve the survival rate.

Mortality rates can reach 32%. Delayed diagnosis and therapy lead to severe complications. In fact, the panesophageal necrosis puts patients at high risk of septic shock and of esophageal perforation through transmural involvement [3]. Approximately 60% of patients recover with conservative treatment. Typically, surgery is reserved for treating esophageal perforation that leads to mediastinitis and abscess formation. Standard surgical approaches may involve procedures such as esophagectomy, decortication, lavage, and delayed reconstruction. However, primary closure of the perforation itself is generally not recommended [13].

In a significant case series, about 25% of patients with AEN developed esophageal strictures and needed endoscopic dilations [2].

## Conclusions

We presented a case of AEN triggered by hypoperfusion following a significant blood loss during surgery. It is a rare but highly fatal condition when diagnosis and therapy is delayed. EGD with careful biopsies are capital to confirm diagnosis. Rapid hemodynamic stabilization with blood transfusion and intravenous fluids, intravenous PPI and parenteral nutrition are the crucial principles for the management of AEN and could guarantee an improved disease outcome.
